# Inferring network model from local field potentials

**DOI:** 10.1186/1471-2202-14-S1-P255

**Published:** 2013-07-08

**Authors:** Julia Makarova, Oscar Herreras, Valeri A Makarov

**Affiliations:** 1 Dept. of Systems Neuroscience, Instituto Cajal, CSIC, Madrid 28002, Spain; 2 Dept. of Applied Mathematics, F. CC. Matemáticas, Universidad Complutense, Madrid 28040, Spain

## 

**Figure 1:**
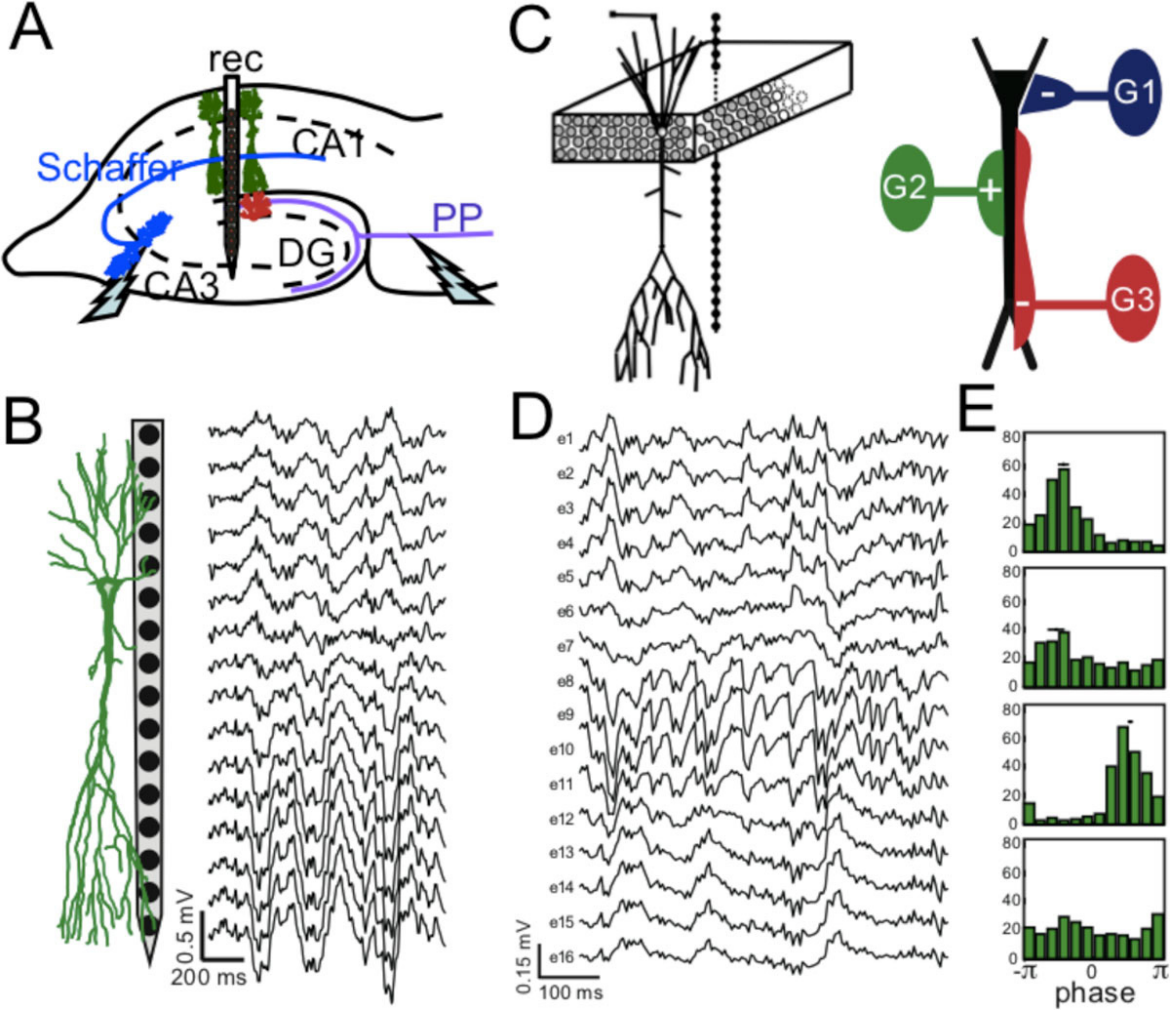
**Inferring network dynamics from LFPs**. **A**. Sketch of experimental setup. Multi-electrode spans CA1-CA3 region of the rat hippocampus. **B**. Experimental spontaneous LFPs. **C**. local circuit included in the model. **D**. Simulated LFPs. **E**. Phase-spike relations.

Experimental study of neural information processing requires simultaneous sampling of the activity of multiple cells over the network. One of the promising approaches relies on monitoring local field potentials (LFPs) by multi-electrode arrays that can span large brain regions (see Figure 1A,B). LFPs may contain firing activity of different cells and, more importantly, they capture synaptic activity produced by principal cells. Thus LFPs enclose extremely rich information on how principal cells integrate converging inputs coming from multiple neuronal populations. However, since synaptic currents mix in the extracellular volume, LFPs have complex spatiotemporal structure making them hard to be exploited. Recently we proposed a biophysical framework to identify and separate LFPs into so-called LFP-generators [1]. Here we discuss how this technique can be used to identify spatial characteristics and temporal patterns of main LFP-generators actuating in the rat hippocampus. The electrical activity of each generator is defined by the spatial distribution of synaptic terminals and the time course of synaptic currents initiated by spike trains sent by the corresponding presynaptic population. Remarkably some recognized pathways do not contribute notably to LFPs [2]. We localized synaptic inputs with subcellular accuracy and reconstructed time courses of main LFP-generators up to millisecond time scales. We were able to readout in parallel the pathway-specific presynaptic activity of projection cells in the entorhinal cortex and pyramidal cells in the ipsilateral and contralateral CA3. We also provide results on spatial extension of the generators. Then we use a computational multi-neuronal model (see Figure 1C) that scales up single cell electrogenesis driven by several synaptic inputs to realistic aggregate LFPs. This approach relies on the fixed but distinct locations of synaptic inputs from different presynaptic populations obtained experimentally. We show that this approach reliably reproduces ongoing LFPs in the hippocampus (see Figure 1D). Then simulated LFPs can be used for studying relationships between phases of LFP-generators and spike trains fired by presynaptic neurons (see Figure 1E). We show that the phase-spike relations provide information on spatial location of axon terminals in contrast to extended opinion on phase spike locking.
